# EyeMatics: An Ophthalmology Use Case Within the German Medical Informatics Initiative

**DOI:** 10.2196/60851

**Published:** 2024-12-05

**Authors:** Julian Varghese, Alexander Schuster, Broder Poschkamp, Kemal Yildirim, Johannes Oehm, Philipp Berens, Sarah Müller, Julius Gervelmeyer, Lisa Koch, Katja Hoffmann, Martin Sedlmayr, Vinodh Kakkassery, Oliver Kohlbacher, David Merle, Karl Ulrich Bartz-Schmidt, Marius Ueffing, Dana Stahl, Torsten Leddig, Martin Bialke, Christopher Hampf, Wolfgang Hoffmann, Sebastian Berthe, Dagmar Waltemath, Peter Walter, Myriam Lipprandt, Rainer Röhrig, Jens Julian Storp, Julian Alexander Zimmermann, Lea Holtrup, Tobias Brix, Andreas Stahl, Nicole Eter

**Affiliations:** 1 Institute of Medical Informatics, University of Münster, Institut für Medizinische Informatik Münster, Albert-Schweitzer-Campus 1, Münster, 48149, Germany; 2 Department of Ophthalmology, University Medical Center Mainz, Mainz, Germany; 3 Department of Ophthalmology, University Medicine Greifswald, Greifswald, Germany; 4 Hertie Institute for AI in Brain Health, Tübingen AI Center, University of Tübingen, Tübingen, Germany; 5 University of Bern, Switzerland, Bern, Switzerland; 6 Institute for Medical Informatics and Biometry, Technische Universität Dresden, Dresden, Germany; 7 Department of Ophthalmology, Klinikum Chemnitz, Chemnitz, Germany; 8 Department of Computer Science, University of Tübingen, Tübingen, Germany; 9 Institute for Bioinformatics and Medical Informatics, University of Tübingen, Tübingen, Germany; 10 Translational Bioinformatics, University Hospital Tübingen, Tübingen, Germany; 11 Department for Ophthalmology, University Eye Clinic, Eberhard Karls University of Tübingen, Tübingen, Germany; 12 Centre for Ophthalmology, University of Tübingen, Tübingen, Germany; 13 Trusted Third Party of the University Medicine Greifswald, Greifswald, Germany; 14 Medical Informatics Laboratory, Institute for Community Medicine, University Medicine Greifswald, Greifswald, Germany; 15 Department of Ophthalmology, University Hospital RWTH Aachen, Aachen, Germany; 16 Institute of Medical Informatics, RWTH Aachen University, Aachen, Germany; 17 Department of Ophthalmology, University Hospital Münster, Münster, Germany

**Keywords:** digital ophthalmology, interoperability, precision ophthalmology, patient engagement, Germany, clinical use, intravitreal, injections, eye, treatment, patient data, framework, AI, artificial intelligence, biomarker, retinal, scan, user-centered, observational

## Abstract

The EyeMatics project, embedded as a clinical use case in Germany’s Medical Informatics Initiative, is a large digital health initiative in ophthalmology. The objective is to improve the understanding of the treatment effects of intravitreal injections, the most frequent procedure to treat eye diseases. To achieve this, valuable patient data will be meaningfully integrated and visualized from different IT systems and hospital sites. EyeMatics emphasizes a governance framework that actively involves patient representatives, strictly implements interoperability standards, and employs artificial intelligence methods to extract biomarkers from tabular and clinical data as well as raw retinal scans. In this perspective paper, we delineate the strategies for user-centered implementation and health care–based evaluation in a multisite observational technology study.

## Introduction

In the burgeoning field of digital health, the necessity for cross-site real-world data and the establishment of medical informatics standards have become increasingly apparent and are key drivers in the nationwide Medical Informatics Initiative (MII) of Germany [1]. The gap between real-world and clinical studies is marked by differences in patient demographics, disease stage, comorbidities, treatment regimens, and outcomes. Clinical trials often operate under controlled conditions with selected patient populations, which may not fully represent the real-world scenario [2]. Medical informatics, using consistently international syntactic and semantic standards for clinical data exchange, bridges this gap by integrating and analyzing diverse, real-world data across multiple sites. This approach enhances the understanding of disease patterns, treatment effectiveness, and patient outcomes in everyday clinical practice, providing a more comprehensive evidence base for health care decision-making.

The EyeMatics module is one of the new clinical use cases in the MII that exemplifies this approach in ophthalmology by harmonizing data from 4 university hospitals and 2 further rollout partners in Germany. The project spans from 2024 to 2028 and aims to connect various ophthalmic information systems to provide a comprehensive and in-depth view of patients who receive intravitreal injections (IVI), one of the most frequent surgical treatments in the outpatient setting with over 1,000,000 procedures conducted per year in Germany [3], for managing conditions such as exudative age-related macular degeneration and diabetic macular edema, which are two of the main causes of blindness in the country [4]. As the population ages, the prevalence of conditions treated with IVI will continue to increase [5], emphasizing the critical role of IVI in preserving vision and addressing a major health care challenge [6].

In this perspective paper, we present the cross-site and cross-state EyeMatics approach, which enhances ophthalmic research by connecting previously isolated subsystems, such as retinal scans through optical coherence tomography (OCT), clinical assessment data, and patient-reported outcomes (PROs). Moreover, we ensure sustainable data exchange by consistently utilizing international interoperability standards. We prioritize patient engagement through the collection of PROs and actively integrating patient representatives into the project governance. By obtaining broad consent (BC) from the patients, rigorously upholding data privacy, and regulating the use of medical devices, we aim to establish digital health tools aligning with strict regulatory frameworks while also enabling innovation in ophthalmic care. EyeMatics is Germany’s largest digital health initiative for eye diseases and could serve as a blueprint for the development of patient-centric nationwide health initiatives in other countries as well; it is supported by significant funding from the Federal Ministry of Education and Research.

The overarching objective of this multipartner, multidisciplinary project is to improve our understanding of the therapeutic success in eye diseases commonly treated with IVI by providing a data exchange and analysis platform of “real-world” clinical data extracted and integrated from and across various data sources and sites. This objective will be achieved by accomplishing the following subobjectives:

- To provide, for the first time, a core dataset of the exchange of medical patients’ data in ophthalmology in order to harmonize cross-consortia data capture and analyses
- To demonstrate a functional privacy-preserving data storage and exchange infrastructure for this dataset
- To integrate functional automated data extracting and merging mechanisms
- To establish a dashboard as a data visualization and analysis platform
- To routinely collect and analyze PROs and to integrate these in the clinical dashboard
- To research biomarkers of ophthalmological assessment and imaging data, which are predictive for disease progression and thus treatment success
- To incorporate results in the guidelines of the German Ophthalmological Society

## Methodology

The following sections present key components of the EyeMatics initiative comprising a governance strategy, technical infrastructure, and health care evaluation, all of which are integral to the overarching goal of implementing and evaluating the cross-state digital health initiative in ophthalmology.

### Patient Population

EyeMatics is planned as an observational health technology study at 4 university hospital sites with the aim to include 2 more hospital sites as the technology study is rolled out. All patients who have undergone inpatient or outpatient procedures with IVI and signed BC forms are going to be included. The consent form is crucial for ethical participation, legal requirements, and data sharing according to the guidelines of the MII. The study employs a longitudinal analysis to track patient health over time, aiming to understand the long-term effects of procedures, patient satisfaction, and possible complications.

### Governance

The governance of the consortium consists of the Management Board (MB) that includes an interplay of patient representatives (n=2); domain experts in clinical ophthalmology, epidemiology, and basic biomedical science (n=6); and experts in biomedical informatics (n=5). It is responsible for the execution of all work packages and key decision-making. The MB is supported by an international external advisory board and other national representative bodies including the National Steering Group and the consortia boards of the MII—to ensure medical informatics standards, particularly on interoperability, data protection, and patient consent—and the German Ophthalmological Society to align project developments with general requirements by domain experts on a national level.

**Figure 1.**
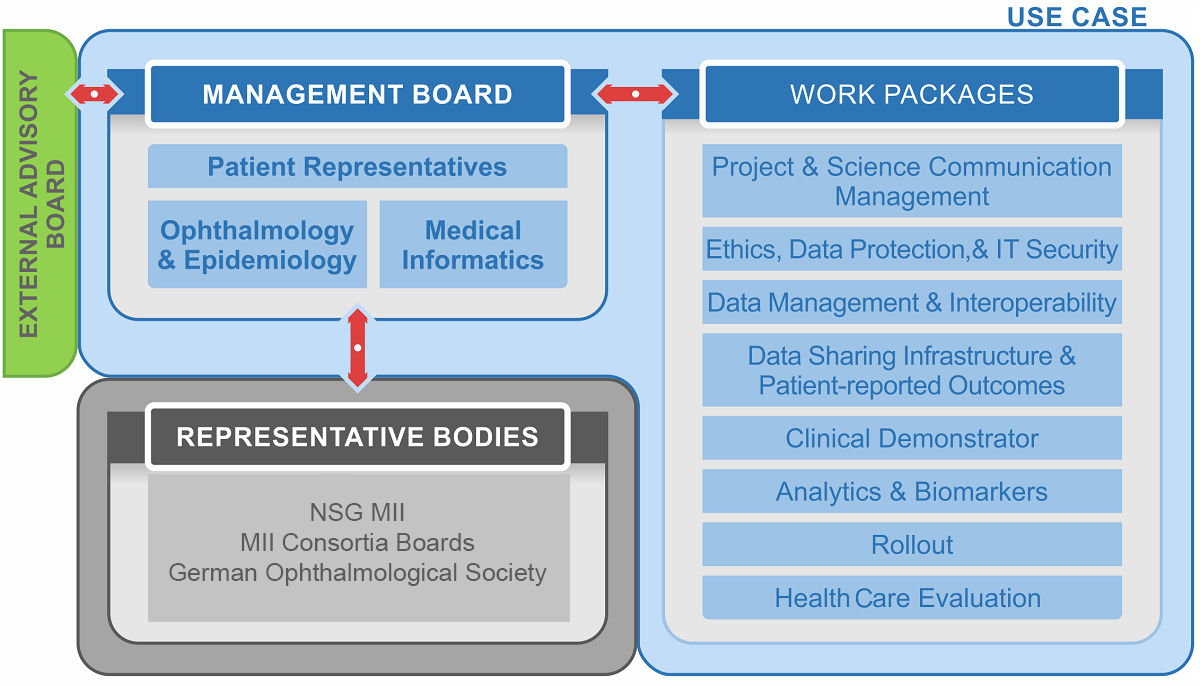
Governance structure. MII: Medical Informatics Initiative; NSG: National Steering Group.

At the core of the MB is the partnership with Pro Retina Deutschland e.V., a not-for-profit self-help association for people with retinal degeneration, from where 2 patient representatives are included. This is essential for establishing science communication and for planning and developing digital tools such as PROs or quality of life indicators in a user-centric way (Figure 1).

### Technical Infrastructure

#### Foundational Infrastructure: The Medical Informatics Initiative

EyeMatics leverages the MII infrastructure to enable the integration of health care data across local data integration centers (DICs) [7] in order to connect medical data across university hospitals and community hospitals. To standardize basic data definitions and data exchange, the MII has established core datasets for transferring medical data. Each DIC within the MII is tasked with extracting these datasets from clinical systems to support medical research inquiries. The MII core datasets are technically defined in Health Level 7 (HL7) Fast Healthcare Interoperability Resources (FHIR) and include several fundamental categories, such as procedures, diagnoses, and laboratory findings, alongside various specialized modules tailored for specific research projects. There is presently no specialized module for ophthalmology that has been agreed upon or is under active consideration by the clinical leadership of the German Ophthalmological Society, and therefore, EyeMatics will establish a dedicated extension module.

A critical aspect of the MII infrastructure is the standardized BC for the long-term secondary use of health data and biosamples, which has been evaluated for General Data Protection Regulation compliance and approved by all relevant data protection authorities in Germany [8]. Each DIC of a participating hospital integrates data from primary information systems and implements the data sharing framework (DSF) to enable decentralized data exchange [9]. For each site, the DSF-FHIR-Server and DSF Business Process Engine enable research data exchange among the DICs. The DSF-FHIR-Server acts as a digital mailbox for data reception, while the DSF Business Process Engine manages further data processing, with the system’s modularity and scalability being enhanced by DSF plug-ins for specific applications, akin to smartphone apps.

#### EyeMatics Infrastructure

EyeMatics is one of the seven clinical use cases that were selected for funding after competitive application to extend the nationwide MII infrastructure. The project utilizes the MII infrastructure and implements the following technical solutions to realize an ophthalmology use case.

##### Ophthalmology Extension Module

Tailored to capture the core dataset definitions pertinent to ophthalmology, the Ophthalmology Extension Module will ensure that data captured are consistent and interoperable across different systems within the EyeMatics project. The dataset will be planned and agreed upon primarily on the basis of clinical research needs by clinical experts from the German Ophthalmology Society and patient representatives. It will be technically specified within the HL7 FHIR standard with value sets and code systems such as Systemized Nomenclature of Medicine–Clinical Terms (SNOMED-CT) and Logical Observation Identifiers Names and Codes (LOINC) to ensure semantic interoperability. The process is further governed by the national steering committee of the MII to ensure alignment with interoperability needs across diseases. The extension module will be versionized and publicly available for reuse and commenting on the portal of Medical Data Models [10] and Art DÉCOR [11]. In the planning phase, the core data elements will be examination items such as tonometry, refraction, visual acuity, medication details, further surgeries (ie, cataract surgery), laboratory data including glycated hemoglobin, and OCT imaging details such as retinal thickness for each eye. These will be extended, further granularized, and technically specified in the first year of the project.

##### Local Data Sharing Infrastructure

**Figure 2.**
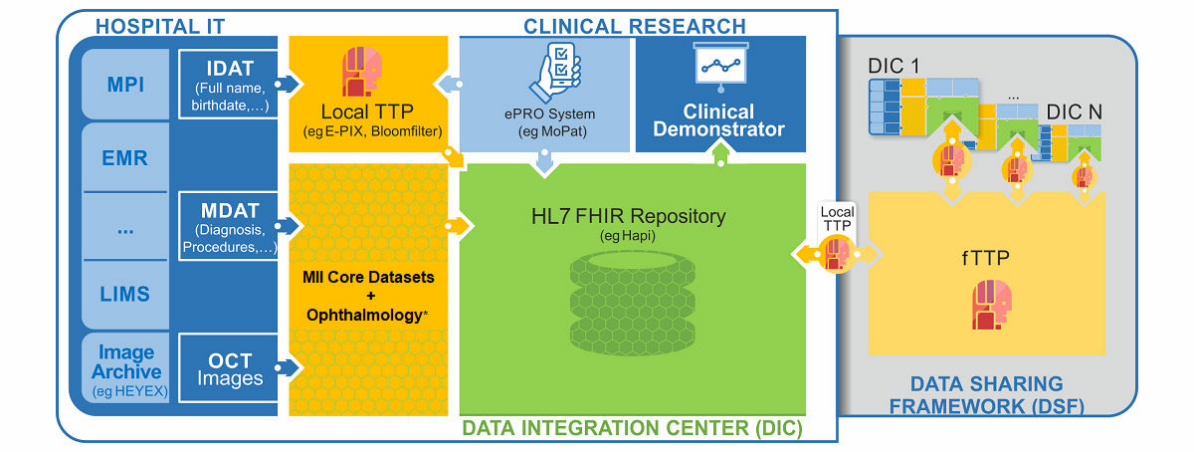
Technical infrastructure of EyeMatics. Following the core dataset specification, data are exported from the primary hospital systems and stored in a local HL7 FHIR repository. Additional ePRO data are collected and are linked by a pseudonym from a local TTP. Data will be exchanged with other sites by using the data sharing framework. A global federated TTP is used for record linkage. The data and analysis results can be visualized in the clinical demonstrator. *In addition to existing nationwide core datasets of the MII, the project will establish an extension module to capture and harmonize data items in the ophthalmology domain. EMR: electronic medical record; ePRO: electronic patient-reported outcome; FHIR: Fast Healthcare Interoperability Resources; HL7: Health Level 7; IDAT: identifiable data; LIMS: laboratory information system; MDAT: medical data; MII: Medical Informatics Initiative; MPI: master patient index; OCT: optical coherence tomography; TTP: trusted third party.

The existing DSF of the basic MII infrastructure will be utilized for data transfer between DICs. Each DIC has already implemented extracts, transforms, and loading procedures for the basic MII core datasets and provides local pseudonymization services. In order to add the ophthalmology extension module, further primary information systems are required to be connected to a project-specific HL7 FHIR repository of the DIC, including data from the electronic medical records, laboratory information system, and the OCT scans from the imaging archive of the ophthalmology department. Pseudonymization and record linkage on a cross-site level will be established by a global federated trusted third party [12]. In a first iteration, each DIC shares and stores the medical data of all DICs in periodic cycles. In further iterations, a caching mechanism and storage of aggregated values will be implemented to reduce data redundancy. The final goal is a real-time data exchange on request. In addition to the DSF, a mobile ePRO service must be installed and configured to link PRO data into the existing FHIR repository. The data items of PRO instruments will be developed as part of the project (see below, section Health Care Evaluation). Figure 2 illustrates the technical architecture within one DIC.

##### Bridging Images and Clinical Data

While a HL7 FHIR repository is employed to ensure a standardized exchange of clinical data, the Digital Imaging and Communications in Medicine (DICOM) standard is used for handling medical imaging information. DICOM plays a crucial role in managing the vast imaging data, similar to OCT, ensuring it is correctly linked to patient records. The strategy of bridging clinical and imaging raw data with HL7 FHIR and DICOM involves exporting DICOM metadata of OCT images into FHIR’s ImagingStudy resources, which will then be stored in the local FHIR repository, allowing for efficient querying of image metadata without the need to store large image files directly in the FHIR repositories. This approach will enable seamless interoperability between the medical imaging and clinical data domains, facilitating queries that link patient diagnoses with available imaging data, and ensuring that image data can be accessed through standardized DICOM services linked within the FHIR resources.

**Figure 3.**
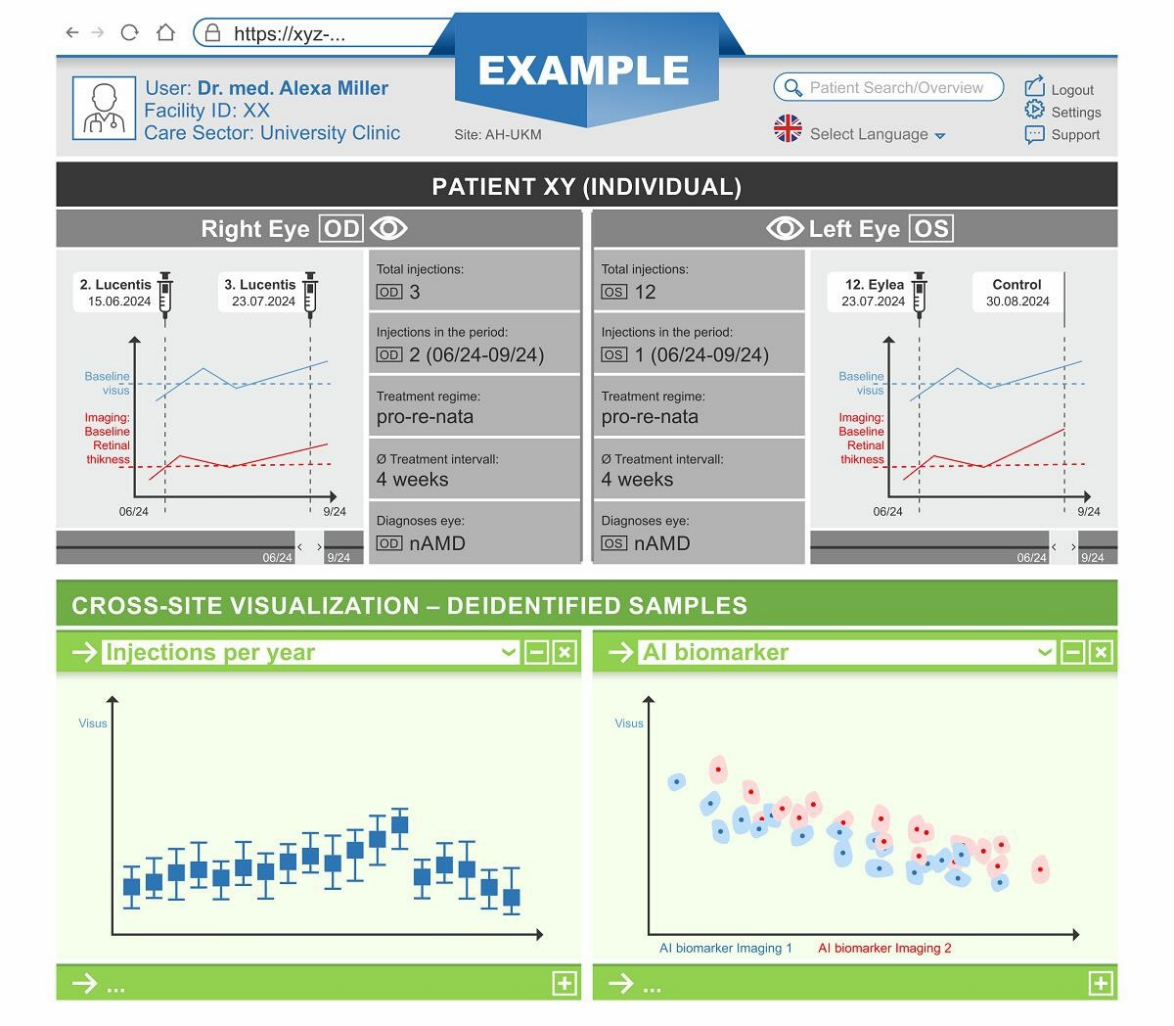
Mock-up of the clinical demonstrator. The cross-site visualization collects deidentified samples across sites via the data sharing framework and the global federated trusted third party. AI: artificial intelligence; nAMD: neovascular age-related macular degeneration.

The clinical demonstrator is an advanced interface that is connected to the local DIC (Figure 3). It visualizes data at the level of individual patients as well as at the cohort level of all partners. The purpose is to present patient information and imaging data in an accessible manner. Individual and aggregated cohort data for comparison need to be visualized separately for the left and right eyes, an essential feature lacking in general electronic medical record systems. A close collaboration and feedback process with clinicians will enable a user-centric design. This especially includes formative and summative usability evaluations. During the project, the demonstrator is not intended as a medical device or clinical decision support as it only features an integrated view of previously isolated data and analyses results. None of its elements, including the artificial intelligence (AI) biomarkers, represent calculations or treatment advice for an individual patient but important associations on a cohort level. However, to prepare individual AI-based treatment predictions, the AI pipeline will already involve regulatory preparations in all the planning and development phases (see AI-Based Biomarkers and European Union Medical Devices Regulations).

##### AI-Based Biomarkers and European Union Medical Devices Regulations

Advanced data analysis aims to discover new imaging-based biomarkers indicative of therapy success by integrating monitoring tools and imaging-based biomarkers into the clinical dashboard. The goal is to create optimized treatment plans as a conceptual demonstration of the AI’s capabilities in precision medicine.

To do this, we will model patient trajectories based on OCT volumes, disease-relevant laboratory data such as glycated hemoglobin, treatment information, and general patient data. We will use deep learning models to extract the disease course of each patient in a latent space and model the trajectories of each patient, especially in response to interventions. The deep learning module will make use of recently published pretrained foundation models such as RetFound [13]. From this latent space, we will predict the evolution of visual acuity as one of the main patient outcomes. Subsequently, we will derive biomarkers for successful treatment by using gradient-based techniques to backpropagate the information about successful treatment to the input data. This way an AI model will be established that is able to optimize treatment plans for specific individual patients.

Within the project duration, the resulting biomarkers will be only utilized for scientific knowledge discovery and not as an interventional medical device for clinical decision-making. However, to prepare for the translation of the information into clinical routines, the relevant steps for the planning and development of the AI algorithms will be accompanied in accordance with European Union Medical Devices Regulation and the upcoming EU AI acts by strictly applying quality management according to EN-ISO 13485, technical documentation of software life cycles, and risk management. To do this, one of the core research partners is already certified according to EN-ISO 13485. Moreover, a medical device vendor will prepare all the necessary documentations along with the planning and development of AI software from the start of the project.

## Health Care Evaluation

Health care evaluation will include the development, implementation, and evaluation of suitable and tailored patient-reported outcome measures (PROMs) in close collaboration with patient representatives as well as analysis of quality indicators.

### Patient-Reported Outcome Measures

Existing PROMs for vision research need to be translated into the German language and validated in a digital self-administered format, as most PROMs are developed as paper-pencil questionnaires. The PROM psychometric performance will be evaluated to obtain validated measures. They need to be formatted in a way to meet the requirements of older adults with low vision, as well as those who are undergoing IVI treatment.

The identification of the relevant aspects of the IVI treatment for patients will be jointly determined with the patient representatives to meet patient aspects. Existing validated questionnaires such as the National Eye Institute 25-Item Visual Functioning Questionnaire [14] that gathers information about the self-reported vision-targeted health status will be the basis for these further developments. This questionnaire analyzes two Rasch-based [15] unidimensional scales, namely the visual-functioning scale and the socio-emotional scale. This will be supplemented by specific questionnaires with respect to adherence aspects and treatment burden to reflect the quality of life aspects of patients receiving IVI treatment. The resulting PROMs will be developed within a tablet-based data capture solution for patients during patient visits.

### Evaluation of Quality Indicators of IVI Treatment

Different aspects of treatment quality will be analyzed including common quality indicators of IVI treatment.

The latency time within the treatment and monitoring cycles will be determined as the days between the indication or OCT examination and performing the IVI treatment within one cycle of treatment. This needs to be adjusted for the different treatment patterns, such as fixed schemes and “treat and extend” schemes.

Treatment and monitoring frequency will be determined for the first 3 months (the upload phase in IVI treatment) and for 12, 24, and 36 months of treatment. The number of IVI treatments and OCT examinations will be determined for each patient as quality indicators and will be stratified on the basis of the treatment pattern, indication, and clinical center.

Nonadherence to treatment will be determined as the number of patients without an intended treatment or monitoring gap of 3 months. Nonpersistence of treatment will be computed as those patients not having any monitoring or treatment of the disease within 6 months and compared for treatment pattern, indication, and clinical center.

As medical outcomes, the change in visual acuity and PROMs over the treatment course will be analyzed. Statistical comparison between the different IVI therapy regimens, indications, and clinical centers (initial clinics, rollouts) using standard statistical descriptive and analytical procedures will be conducted. Data will be compared to regulatory studies and other “real-world” studies. Time-to-event analysis in case of nonadherence or nonpersistence will be carried out. Mixed-model approaches will be used to incorporate one or two treated eyes of one patient.

## Expected Outcomes

On the technical and research front, the EyeMatics project is set to significantly enhance the interoperability of ophthalmology data through the semantic integration of imaging metadata, particularly from OCT, into the DICOM and FHIR standard with existing modules from the MII. We thereby streamline the sharing and utilization process of ophthalmology data across diverse platforms and institutions. The newly established data foundation will help to develop and test mechanistic and novel deep learning approaches to identify imaging biomarkers, bridging the gap between various health parameters, ocular surfaces, and the effectiveness of therapeutic interventions.

The implementation of the BC—forming the legal basis—at the participating sites will ensure direct added value not only for the EyeMatics project but also for other medical research projects.

Moreover, on the health care outcome side, the project is expected to yield validated PROMs specifically adapted for ophthalmology, particularly for patients undergoing IVI treatments. These PROMs are designed to be digitally self-administered, catering to the unique needs of older adult patients with low vision. The insights garnered from these PROMs are combined with the interoperable data framework and AI-derived diagnostic biomarkers in a special dashboard and made available to the treating physicians on a pilot basis. This holistic approach promises to not only refine clinical practices but also foster personalized patient care by incorporating real-time data evaluation and patient-centric outcomes into therapeutic decision-making processes. The results of the project will contribute to the adaptation of more nuanced and effective guidelines for IVI treatment and help to strengthen treatment adherence and persistence in the face of demanding treatment regimens. In the long term, this should lead to an improvement in treatment outcomes.

## Abbreviations

AI: artificial intelligence
BC: broad consent
DIC: data integration center
DICOM: Digital Imaging and Communications in Medicine
DSF: data sharing framework
FHIR: Fast Healthcare Interoperability Resources
HL7: Health Level 7
IVI: intravitreal injections
LOINC: Logical Observation Identifiers Names and Codes
MB: Management Board
MII: Medical Informatics Initiative
OCT: optical coherence tomography
PRO: patient-reported outcome
PROM: patient-reported outcome measure
SNOMED-CT: Systemized Nomenclature of Medicine–Clinical Terms
